# The Bacterial Phytoene Desaturase-Encoding Gene (*CRTI*) is an Efficient Selectable Marker for the Genetic Transformation of Eukaryotic Microalgae

**DOI:** 10.3390/metabo9030049

**Published:** 2019-03-12

**Authors:** Ana Molina-Márquez, Marta Vila, Javier Vigara, Ana Borrero, Rosa León

**Affiliations:** 1 Laboratory of Biochemistry, Faculty of Experimental Sciences, Marine International Campus of Excellence (CEIMAR), University of Huelva, 2110 Huelva, Spain; ana.molina@dqcm.uhu.es (A.M.-M.); mvila@phycogenetics.com (M.V.); vigara@uhu.es (J.V.); anika_b92@hotmail.com (A.B.); 2 PhycoGenetics SL, C/Joan Miró Nº6, Aljaraque, 21110 Huelva, Spain

**Keywords:** microalgae, *Chlamydomonas reinhardtii*, genetic transformation, carotenoid, *CRTI*, phytoene desaturase

## Abstract

Genetic manipulation shows great promise to further boost the productivity of microalgae-based compounds. However, selection of microalgal transformants depends mainly on the use of antibiotics, which have raised concerns about their potential impacts on human health and the environment. We propose the use of a synthetic phytoene desaturase-encoding gene (*CRTIop*) as a selectable marker and the bleaching herbicide norflurazon as a selective agent for the genetic transformation of microalgae. Bacterial phytoene desaturase (CRTI), which, unlike plant and algae phytoene desaturase (PDS), is not sensitive to norflurazon, catalyzes the conversion of the colorless carotenoid phytoene into lycopene. Although the expression of *CRTI* has been described to increase the carotenoid content in plant cells, its use as a selectable marker has never been testedin algae or in plants. In this study, a version of the *CRTI* gene adapted to the codon usage of *Chlamydomonas* has been synthesized, and its suitability to be used as selectable marker has been shown. The microalgae were transformed by the glass bead agitation method and selected in the presence of norflurazon. Average transformation efficiencies of 550 colonies µg^−1^ DNA were obtained. All the transformants tested had incorporated the *CRTIop* gene in their genomes and were able to synthesize colored carotenoids.

## 1. Introduction

Microalgae have attracted considerable interest for the production of a wide range of compounds of applied interest due to their easy growth, their ability to fix atmospheric CO_2_, and the valuable metabolites that some species can produce, which include pigments, food supplements, vitamins, antioxidants, polysaccharides, lipids, and other bioactive products [1,2,3]. Moreover, in the last years, there has been an increasing interest in microalgae as a potential source of biofuels [4,5]. However, despite the expectations generated, the production of biofuels and other useful compounds from microalgae will not be economically feasible unless the cost of microalgae cultivation and harvesting is lowered and the productivity increased. Genetic manipulation and synthetic biology show great promise to further boost the productivity of microalgae-based compounds [6,7,8,9]. Significant advances have been achieved in the development of molecular tools for genetic manipulation of microalgae. However, important challenges remain. One important issue is the fact that the selection of microalgal transformants depends mainly on the use of antibiotics as selective agents [10]. Antibiotic resistance genes continue to be the most commonly used selectable markers for the genetic manipulation of algae and plants. However, the risk of horizontal gene transfer has raised concerns about their potential impacts on human health and the environment, and has encouraged the search for new non-antibiotic-based selection procedures [11].

Herbicide resistance genes are a good alternative for the selection of genetically modified plant cells. Examples of this are the glyphosate aminotransferase [12] or the acetolacetate synthase genes [13], which confer resistance to glyphosate and sulfomturon methyl herbicides, respectively, and have been used as reporter genes in the transformation of the unicellular chlorophyte *Chlamydomonas reinhardtii* and other microalgae, such as *Porphyridium* sp. [14] or *Parietochloris incisa* [15]. An interesting herbicide-based selective strategy is the use of mutated versions of the phytoene desaturase gene (*PDS*) resistant to bleaching herbicides such as norflurazon [16,17]. Phytoene desaturase (PDS) catalyzes the conversion of the colorless phytoene into ζ-carotene, which is converted to lycopene by ζ-carotene desaturase (ZDS) and carotene isomerase (CRTISO). PDS is a membrane-associated protein that uses the flavin adenine dinucleotide (FAD) as a redox cofactor, through which electrons are transferred to the plastoquinone, thereby connecting the desaturation of carotenoids with the photosynthetic electronic transport chain [1]. Treatment with norflurazon causes inhibition of phytoene desaturase by competition with its cofactors, resulting in suppression of carotenoid synthesis and cellular whitening [18]. By modifying key amino acids in the FAD binding domain, some authors have obtained mutated norflurazon-resistant versions of PDS and setup genetic selective procedures based on norflurazon as a selective agent [17,19].

In bacteria and fungi, however, the three reactions that convert phytoene into lycopene are carried out by a single enzyme, bacterial phytoene desaturase (CRTI), which presents a low degree of homology with the corresponding plant phytoene desaturase [20]. The *CRTI* gene seems to have emerged independently in evolution and, unlike plant and algae PDS, is not sensitive to norflurazon [21]. The *CRTI* gene was first identified and functionally analyzed by Misawa and coworkers [22] from the soil bacteria *Erwinia uredovora*, currently renamed as *Pantotea ananatis*. The pioneering work of Sandman’s group showed that expression of this bacterial *CRTI* gene in tobacco plants enhanced the production of β-carotene. Furthermore, they observed higher resistance to the herbicide norflurazon in transgenic *CRTI*-expressing plants [23]. Several subsequent studies reported the expression of the bacterial carotenoid gene cluster from *Pantotea*, including the *CRTI* gene, to increase the carotenoid content in higher plants such as rice [24], tomato [25], or potato [26]. However, the possible use of *CRTI* as a selectable marker gene has never been investigated in algae or in plants. Poor expression of bacterial *CRTI* in microalgae, beside potential silencing and lack of stability of the transgene in algae, has withdrawn its use as a selectable marker. In the present study, we have synthesized a codon-adapted version of the bacterial *CRTI* gene and showed its suitability to be used as selectable marker gene in the genetic transformation of the model microalga *Chlamydomonas reinhardtii*.

## 2. Results and Discussion

### 2.1. Construction of Plasmid pSI06PLK-CRTIop

**Figure 1:**
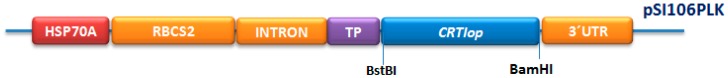
Expression cassette of the pSI106PLK-tp*CRTIop* plasmid.Abbreviations: HSP70A, heat shock protein 70A promoter; RBCS2, ribulose 1,5-biphosphate carboxylase small subunit promoter; TP, chloroplastic transit peptide; *CRTIop*, codon-optimized bacterial phytoene desaturase; 3′UTR terRBCS2, ribulose 1,5-biphosphate carboxylase small subunit terminator region.

A synthetic *CRTI* gene with the codon usage adapted to *C. reinhardtii* was designed and synthesized by Genescript Co (Piscataway, NJ, USA). The mean difference between codon usage of the wild type *CRTI* gene and the genome of *C. reinhardtii* was 24%, as calculated using the graphical codon usage analyser v. 2.0 (http://gcua.schoedl.de/index.html). This difference was reduced to only 13% for the synthetic codon-optimized version. The *CRTIop* gene was fused to a DNA fragment which encoded the chloroplast transit peptide of the RuBisCo small subunit, and this tp*CRTIop* fusion product was cloned between the *BstBI*/*BamHI* restriction sites of the p106PLK plasmid, described in Section 3.2, generating the expression cassette outlined in Figure 1.

### 2.2. Sensitivity of the Chlorophyte Microalgae to the Bleaching Herbicide Norflurazon

**Figure 2:**
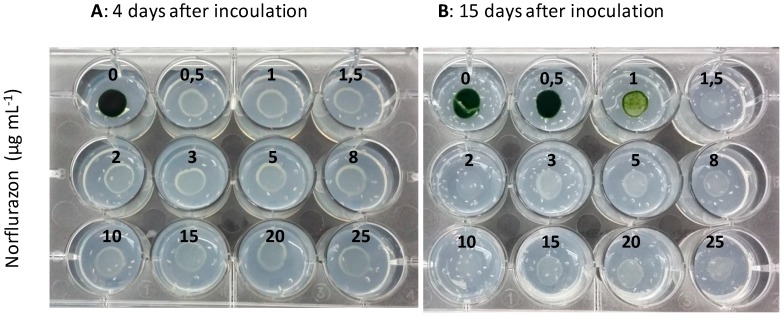
Norflurazon sensitivity test for the clorophyceae microalga *Chlamydomonas reinhardtii*. Drops (10 µL) of a 100-fold concentrated *C. reinhardtii* control untransformed culture were spotted on agar-solidified Tris-acetate phosphate (TAP) culture medium with increasing concentrations of the bleaching herbicide norflurazon, and their growth was evaluated 4 (**A**) and 15 (**B**) days after inoculation.

The ability of *CRTI* to act as a selectable marker for microalgae transformation is based on the sensitivity of the target microalgae to herbicides which inhibit phytoene desaturase (PDS). We have tested the sensitivity of the model chlorophyte *Chlamydomonas reinhardtii* to the bleaching herbicide norflurazon by culturing it with growing concentrations of the herbicide and determining the lethal doses (Figure 2). Samples of control non-transformed *C. reinhardtii* cultures were harvested at the exponential phase of growth and 100-fold concentrated by centrifugation, and 10 µL drops of the concentrated suspension were spotted on multi-well plates with growing concentrations (0–25 µg mL^−1^) of the herbicide norflurazon. Concentration of the culture was done to mimic the conditions in which the transformation experiments are done (see Section 3.3). The minimal inhibitory norflurazon concentration for *C. reinhardtii* was 1.5 µg mL^−1^ after 15 days of incubation in the presence of norflurazon, as can be observed in Figure 2B. It is interesting to note that at shorter times, herbicide concentrations as low as 0.5 µg mL^−1^ seemed to inhibit *C. reinhardtii* growth. However, the microalgaeare able to survive and grow after an adaptation period. To establish the real lethal dose and avoid false negatives, in the subsequent transformation experiments it is necessary to follow the inhibitory effect of norflurazon for a long time period. All *C. reinardtii* transformants were selected at norflurazon concentrations ≥1.5 µg mL^−1^.

Furthermore, sensitivity experiments were carried out for other microalgal species, such as the freshwater trebouxiophyceae *Chlorella sorokiniana*, the marine prasinophyceae *Tetraselmis suecica*, and the halophilic chlorophyceae *Dunaliella salina* and *Dunaliella bardawill*. These studies revealed that the inhibitory norflurazon concentration dose was between 0.5 and 2 µg mL^−1^ for all the tested species, and that norflurazon can be an adequate selective agent for many different microalgal species, including marine microalgae, for which traditional selective antibiotic agents are usually inefficient due to interference with the saline concentration of the medium (Figure S1).

### 2.3. Transformation of Chlamydomonas reinhardtii with the Plasmid pSI106PLK-tpCRTIop and Selection of Norflurazon-Resistant Norf^R^-Chlamydomonas Transformants

**Figure 3:**
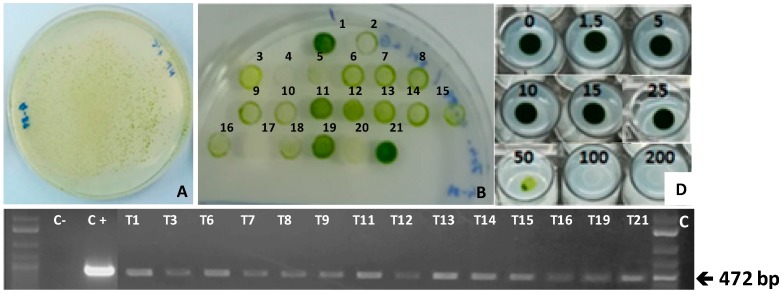
Molecular and phenotypic analysis of Norf^R^-*Chlamydomonas* transformants. *Chlamydomonas* transformants selected in TAP with 1.5 µg mL^−1^ of norflurazon (**A**) were cultured in TAP agar plates with 4 µg mL^−1^ of norflurazon for 10 days (**B**). A band of the expected size (472 bp) corresponding to the *CRTIop* amplicon was shown in all the transformants tested (**C**). A norflurazon sensitivity test for the selected transformant T21 (**D**) was carried out as described in the Figure 2 legend with the indicated concentrations of norflurazon (from 0 to 200 µg mL^−1^).

*Chlamydomonas reinhardtii* cells were transformed by the glass beads agitation method with the plasmid pSI106PLK-tp*CRTIop* and selected in Tris-acetate phosphate (TAP) medium with norflurazon (1.5 µg mL^−1^). Transformation efficiencies of 550 colonies µg^−1^ DNA were obtained (Figure 3A). This transformation efficiency is of the same order as that usually obtained for transformations with paromomycin as the selective agent [27]. A randomly selected group of the obtained transformants were cultured in 2 mL of liquid TAP medium with norflurazon for 48h, and their cellular density was then adjusted to the same value. Drops of each transformed culture were spotted on TAP agar plates with a higher concentration of norflurazon (4 µg mL^−1^), and those which grew at this concentration of the herbicide were further checked by PCR using the specific primers CRTIopFor (CAGCCGCGCCGTGTTCAAAGAG) and CRTIpoRev (CAGCAGGTCGCGGTAGGTGTG), as illustrated in Figure 3B. It is necessary to consider that nuclear transformations of microalgae take place by heterologous recombination. This means that the *CRTIop* gene is randomly inserted into the algal genome, and its expression level and stability largely depend on the insertion point. The two-round selection strategy with increasing concentrations of norflurazon allows the selection of the transformants with the highest resistance to the herbicide. Insertion of the *CRTIop* into the *C. reinhardtii* nuclear genome was confirmed in all the transformants checked (Figure 3C).

The transformants which grew more vigorously were subjected to a norflurazon sensitivity test as described in Figure 1. *C. reinhardtii* transformed with *CRTIop* showed a 33-fold increase in their tolerance to norflurazon, with an inhibitory dose of 50 µg mL^−1^, equivalent to 160 µM, as shown for transformant T21 (Figure 3D) and for the other Norf^R^-transformants selected (Figure S2). The synthetic tp*CRTIop* gene allowed for norflurazon-based selection of transformants with no background of spontaneous herbicide-resistant clones. The level of resistance to norflurazon acquired by the *C. reinhardtii* transformed with *CRTIop* is of the same order as the resistance reported by Suarez et al. [28] or Bruggeman and coworkers [12], who found 30- and 40-fold increases, respectively, in the tolerance to norflurazon for transgenic *Chlamydomonas* harbouring modified versions of its own *PDS* gene. Similar strategies using mutated PDS versions and norflurazon have been successfully used for the selection of other transformed microalgae species, such as *Haematococcuspluvialis* [29,30], *Chlorella zofingiensis* [31,32], or *Isocrhrysis* [33].

### 2.4. Carotenoid Composition of Norflurazon-Resistant Norf^R^-Chlamydomonas Transformants

The phenotypic characteristics of the selected transformants were further studied by chromatographic analysis. First, we compared the phytoene contents in the Norf^R^-transformants with that of the control cells grown in the presence and in the absence of norflurazon (Table 1). As expected, in *C. reinhardtii* cultures grown without the herbicide, only trace levels of phytoene were found. After 24h of growth in the presence of norflurazon (1.5 µg mL^−1^), the phytoene contents in control untransformed cells reached 4.4 mg g^−1^ DW. However, the phytoene intracellular concentrations of Norf^R^-transformants grown with and without norflurazon ranged between 1.5 mg g^−1^ DW, for transformant T21, to 2.1 mg g^−1^ DW, for transformant T11. This is far from the intracellular level of phytoene in the control, which is between two and three times higher. This shows that the *CRTIop* gene is correctly expressed in the transformed microalgae and is able to convert phytoene into the downstream carotenoids. However, the presence of certain contents of phytoene in all the transformants tested indicates that the foreign CRTI is less efficient than the endogenous PDS in the absence of the herbicide.

**Figure 4:**
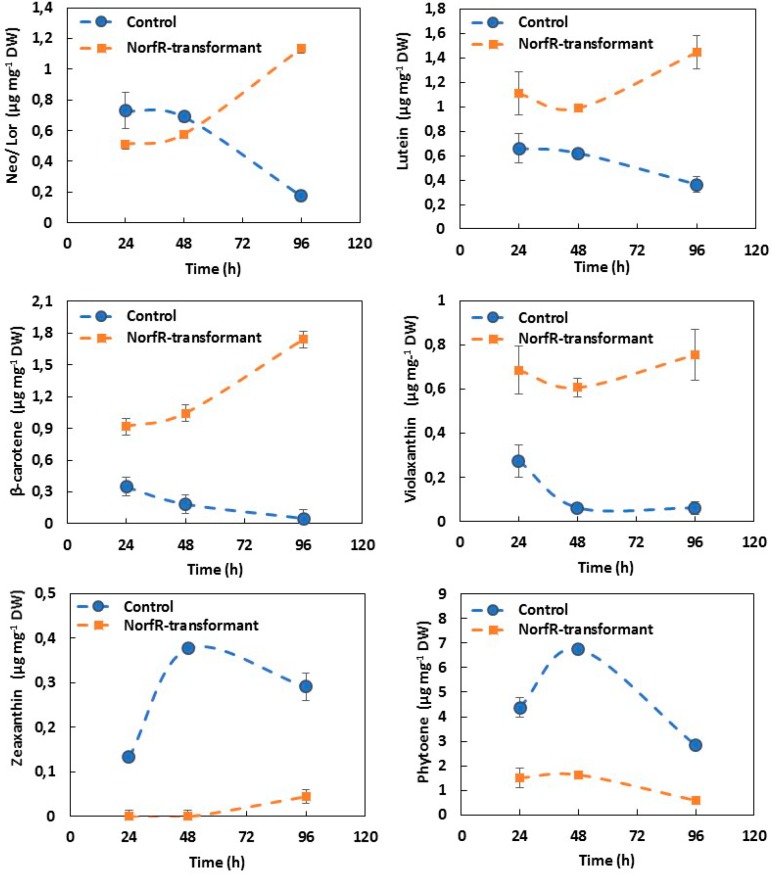
Time-course evolution of the main carotenoid pigments in *C. reinhardtii* control (●) and Norf^R^-transformant (■) cells incubated with norflurazon (1.5 µg mL^−1^). Values are the average of three biological replicates and bars indicate standard deviation. Neoxanthin and Loroxanthin (Neo/Lor) are expressed as unique values since they are not resolved in the analytical conditions used.

A representative Norf^R^-transformant (T21) and the control untransformed strain were grown in TAP medium with norflurazon (1.5 µg mL^−1^) for a complete analysis of their carotenoid profiles along the time. Samples were withdrawn every 24h, and pigments were extracted and analyzed (Figure 4). Typical chromatograms of the pigment extracts from Control (C2) and Norf^R^-transformant cells registered at 288 and 450 nm, and are shown in the Supplementary Material (Figure S3).

In control parental *Chlamydomonas* treated with norflurazon, there was a reduction of all the colored carotenoids, excepting zeaxanthin, and an important accumulation of phytoene, due to the inhibitory effect of norflurazon. By contrast, in transformant cells treated with norflurazon, the reduction of the colored carotenoids and the accumulation of phytoene was much lower. After 24 h of incubation with norflurazon, the content of lutein in the Norf^R^-transfomant was 50% higher than in the controls, while the content in β-carotene and violaxanthin was 2.5 times the level of control untransformed cells. These differences were even more acute for longer periods of incubation with norflurazon. The level of phytoene, on the contrary, was between three and five times higher in the norflurazon-treated control cells, reaching intracelullar levels of 4.4 mg g^−1^ DW at 24 h and 6.7 mg g^−1^ DW at 48h. This confirms that the *CRTIop* gene was working in the transformant, allowing the conversion of phytoene into lycopene and the subsequent carotenoids in the presence of norflurazon, which inhibits the endogenous PDS. Zeaxanthin was the only colored carotenoid which increased in the control untransformed cells, indicating a higher level of fotooxidative stress in these cells. This induces the xantophyll cycle, which catalyzes the conversion of violaxanthin into zeaxanthin. In the Norf^R^-transfomants, the levels of zeaxanthin were inappreciable during the first 48 h. Only after 72 h of growth, when cultures started to be nutrient-limited, there was certain synthesis of this xanthophyll.

In the presence of norflurazon, the growth of the parental strain was severely affected. Meanwhile, the growth rate of the Norf^R^-transformantswas similar with and without norflurazon, and was slightly higher than the growth rate of the control wild type without herbicide (Figure S4A,B). The carotenoid content of the Norf^R^-transformants grown without herbicide was also checked and resulted to be very similar to that of control cells (Figure S4C,D). This means that the transformants selected in the presence of norflurazon with *CRTI* as a marker gene can grow at a normal rate and have practically normal contents of carotenoids [19,20]. It could be expected that the expression of an exogenous phytoene desaturase caused an increase in the contents of carotenoids. The fact that the *CRTIop* transformants studied had intracellular levels similar to that of the control cells in the absence of norflurazon could be due to a limitation in the supply of precursors from the previous step catalyzed by the Phytoene synthase (PSY). However, further studies should be done to confirm this issue.

PDS is the second step in carotenoid biosynthesis and an important regulatory point of the pathway [19,20]. The inhibitory effect of norflurazon is well known and has been widely used to study the physiological consequences resulting from the lack of carotenoids in higher plants and microalgae [34]. We have corroborated that blocking PDS activity by chemical inhibition with norflurazon impedes the formation of downstream carotenoids in *Chlamydomonas* (Figure 4), which is in agreement with what Nigoyi’s group observed by mutagenesis-induced PDS inactivation [34]. Furthermore, we demonstrated that the expression of the foreign *CRTIop*, which is not affected by the herbicide, allows bypass, at least partially, of the norflurazon-blocked step and enables the synthesis of colored carotenoids. Similar conclusions were reported by Liu and coworkers [35] or Steinbrenner and Sandmann [29], who used a modified *PDS* gene as a selectable marker for the genetic transformation of *Chlorella zofingiensis* and as *H. pluvialis*, respectively, and found that the transformants had the same or even higher carotenoid content than the untransformed controls.

## 3. Materials and Methods

### 3.1. Strains and Culture Conditions

*Chlamydomonas reinhardtii* 704 strain (Cw15, Arg7, mt+) was kindly donated by Dr. R. Loppes and cultured photomixotrophically in liquid or agar-solidified Tris-acetate phosphate (TAP) medium [36]. *Tetraselmissuecica*, kindly provided by IFAPA-Aguas del Pino station (Huelva, Spain), was cultured in F/2 medium in filtered sea water at pH 8, as reported by Guillard and Ryther [37]. *Dunaliella salina* (CCAP 19/18) was obtained from the culture collection of algae and protozoa (Scotland, UK) and grown in the culture medium described by Johnson and coworkers [38]. All were grown in a culture chamber at 25 °C under continuous white light irradiation (50 μE m^−2^ s^−1^ PAR).

### 3.2. Microalgal Expression of pSI106PLK Plasmid

Plasmid pSI106PLK is a renewed version of plasmid pSI104PLK [39]. It contains an expression cassette in which a multiple cloning site is preceded by the strong chimeric fusion promoter *HSP70A:RBCS2*, designed by Sizova [40], and the first intron of the *RBCS2* gene, and is terminated by the 3′ untranslated region of *RBCS2* (Figure 1).

### 3.3. Chlamydomonas Nuclear Transformation

Nuclear transformation of *C. reinhardtii* was carried out using the glass beads method [41] with minor modifications. *Chlamydomonas* cultures were grown as described in Section 3.1. to a cell density of 5 × 10^6^ cells mL^−1^, and resuspended to get a 100-fold concentrated cell suspension. 0.3 g of sterile glass beads (0.4–0.6mm Ø), were added to 0.6 mL of concentrated cell suspension with 0.2 mL of 20% PEG (MW8000) and about 1 µg of the desired plasmid (pSI106PLK-tpCRTIop). Negative controls, done in the same conditions with the empty plasmid (pSI106PLK), were included in all the transformation reactions. This mixture was agitated for 10s, resuspended in fresh TAP medium, and spread onto the selective solid medium with the indicated concentration of norflurazon. Transformed colonies were visible after 4 or 5 days.

### 3.4. Determination of Carotenoids

Samples from *C. reinhardtii* cultures, grown in TAP liquid medium supplemented with 1.5 µg mL of norflurazon in the same conditions described in Section 3.1., were used for the extraction of carotenoids with methanol as described by Linchtehaler [42]. The chromatographic analysis was performed in a Merck Hitachi HPLC equipped with a diode array detector as described by Young and coworkers [28] using an RP-18 column, a flow rate of 1mL min^−1^, and a final injection volume of 100 µL. Two mobile phases were used: Solvent A (ethyl acetate 100%) and solvent B (acetonitrile:H_2_O; 9:1 *v*/*v*). The gradients applied were: 0–16 min 0–60% A; 16–30 min 60% A; and 30–35 min 100% A. Standards were supplied by DHI (Hoersholm, Denmark). All experiments were done in triplicate, and the average values and standard deviation are represented. The significant differences have been analysed by a t-student test with a confidence level of 95%.

### 3.5. Dry Weight Determination

Dry weight was determined by filtering an exact volume of microalgae culture (30 mL) on pre-tared glass-fiber filters (GF/F Whatman). The filter was washed with a solution of ammonium formate (0.5 M) to remove salts and dried at 100 °C for 24 h. The dried filters were weighed in an analytical balance and the dry weight calculated by the difference. Values are the average of three measurements.

### 3.6. Herbicide Sensitivity Test

Norflurazon sensitivity was assayed on multi-well plates with the corresponding agar-solidified medium supplemented with the indicated concentrations of the herbicide.

## 4. Conclusions

The use of a synthetic codon-adapted *CRTIop* gene fused to a chloroplastic transit peptide as a selectable marker for the genetic transformation of *Chlamydomonas reinhardtii* has been shown to be a reliable and efficient approach for the selection of transformants, contributing to increase the non-antibiotic-dependent markers available for microalgae and plant cells. *C. reinhardtii* transformants selected on norflurazon have been shown to have the *CRTIop* gene correctly inserted into their genomes to acquire a 33-fold increased resistance to norflurazon and be stable for long periods of time. This is a good alternative to genetic markers based on resistance to antibiotics, which can be very useful to establish genetic transformations systems for new microalgal species which are recalcitrant to inhibition with traditional antibiotics, as usually happens with marine and halophyllic microalgae. It can also be an interesting alternative for the selection of higher plants transformants without using antibiotics.

## Figures and Tables

**Table 1:** Concentration of the colorless carotenoid phytoene in control cells (C2) and Norf^R^-transformants (T), grown for 24h with norflurazon. Control cells non-treated with norflurazon (C1) have also been included as a reference. Values are the average of three biological replications. Standard deviation is indicated.

Strain	C1	C2	T1	T11	T19	T21
Norflurazon (µg mL^−1^)	-	1.5	1.5	1.5	1.5	1.5
Phytoene (µg mL^−1^)	0.1 ± 0.5	4.4 ± 0.4	1.8 ± 0.1	2.1 ± 0.2	2 ± 0.2	1.5 ± 0.1

## Supplementary Materials

The following are available online at https://www.mdpi.com/2218-1989/9/3/49/s1. Figure S1: Norflurazon sensitivity test for several microalgae species. Drops (10 µL) of concentrated cultures of the microalgae *Tetraselmis suecica*, *Dunaliella salina*, *Dunaliella bardawill* and *Chlorella sorokiniana* were spotted on agar-solidified culture medium with increasing concentrations of the bleaching herbicide norflurazon (from 0 to 5 µg mL^−1^) and their growth was evaluated 10 days after inoculation. The culture media used was that described in Materials and Methods for each species, excepting that the concentration of NaCl was reduced to half the salinity of the seawater for *T. suecica* and to 0.5M for *D. salina* and *D. bardawill.* Figure S2: Norflurazon sensitivity test for different Nor^R^-transformants of the microalga *Chlamydomonas reinhardtii*. Drops (10 µL) of concentrated cultures of the transformants T1, T11 and T19 obtained as described in the legend of Figure 2, were spotted on agar-solidified TAP culture medium with increasing concentrations of the bleaching herbicide norflurazon (from 0 to 200 µg mL^−1^) and their growth was evaluated 10 days after inoculation. Figure S3: Chromatographic analysis of pigments extracts from control and Norf^R^-*Chlamydomonas* transfomants grown in the presence of norflurazon (1.5 µg mL^−1^). Conditions for chromatographic separation are described in Materials and Methods. Peaks were identified as: neoxanthin/loroxanthin (1), violaxanthin (2), antheraxanthin (3), lutein (4), zeaxanthin (5), chlorophyll b (6), chlorophyll a (7) and β-carotene (8) and phytoene (9). Figure S4: Growth curves (A,B) and carotenoid content (C,D) of untransformed (Control) and transformed (Norf^R^) strains of *Chlamydomonas reinhardtii* cultured without and with increasing concentrations of the herbicide norflurazon. Cultures of the control untransformed cells and of the Norf^R^-transformant T21 were harvested at the beginning of exponential phase of growth and resuspended in fresh TAP medium without norflurazon or supplemented with 1.5, 8 and 20 µg mL^−1^ of norflurazon. Dry weight (DW) was determined every 24h. In addition, the carotenoid content of control and transformed (Norf^R^) cells grown for 24h without or with 1.5 µg mL^−1^ of norflurazon were also determined. Neoxanthin and loroxanthin (Neo/Lor) are expressed a unique value since they are not resolved in the analytical conditions used. All the values are the average of three biological replicates and bars represent standard deviation.
