# Supplementary material for: The Bacterial Phytoene Desaturase-Encoding Gene (CRTI) is an Efficient Selectable Marker for the Genetic Transformation of Eukaryotic Microalgae
Source: Metabolites. 2019 Mar 12;9(3):49. doi: 10.3390/metabo9030049 (PMC6468381; doi:10.3390/metabo9030049)
Supplement: Supplementary file 1 [file metabolites-09-00049-s001.zip › metabolites-451948-supplementary.pptx]

## Slide 1
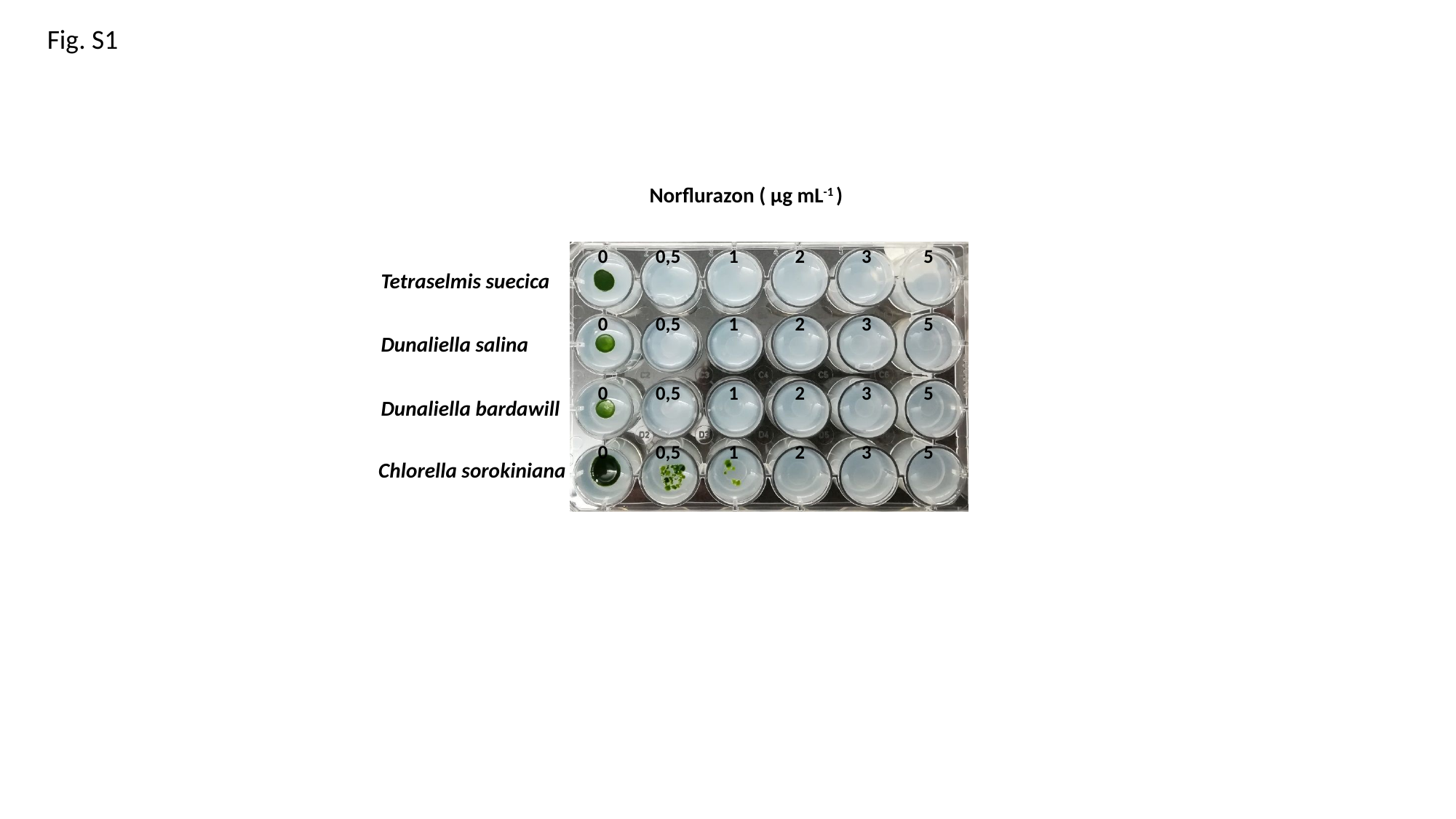

Fig. S1
Norflurazon ( µg mL-1 )
0 0,5 1 2 3 5
0 0,5 1 2 3 5
0 0,5 1 2 3 5
0 0,5 1 2 3 5
Tetraselmis suecica
Dunaliella salina
Dunaliella bardawill
Chlorella sorokiniana

## Slide 2
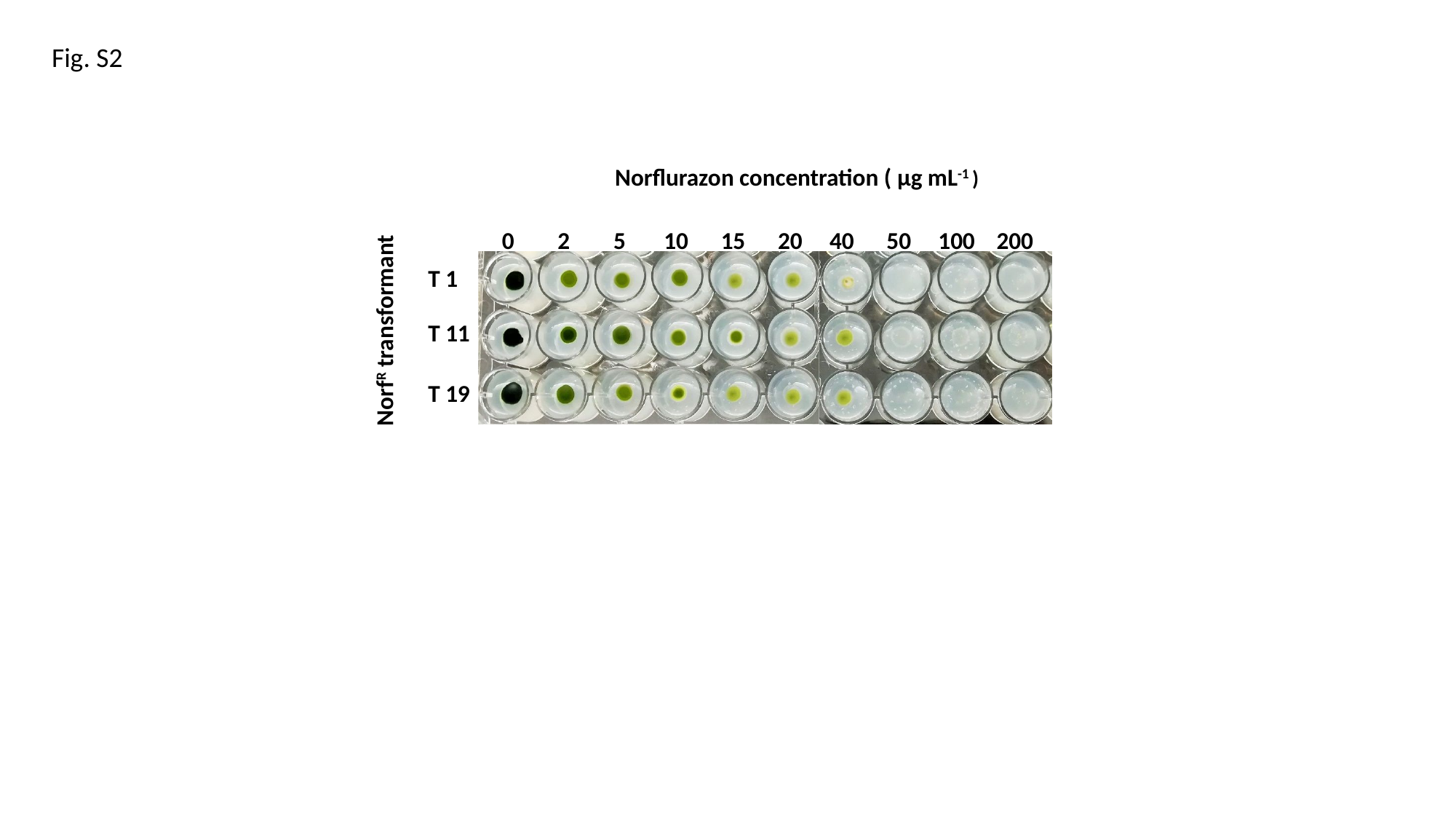

Fig. S2
Norflurazon concentration ( µg mL-1 )
 0 2 5 10 15 20 40 50 100 200
T 1
T 11
T 19
NorfR transformant

## Slide 3
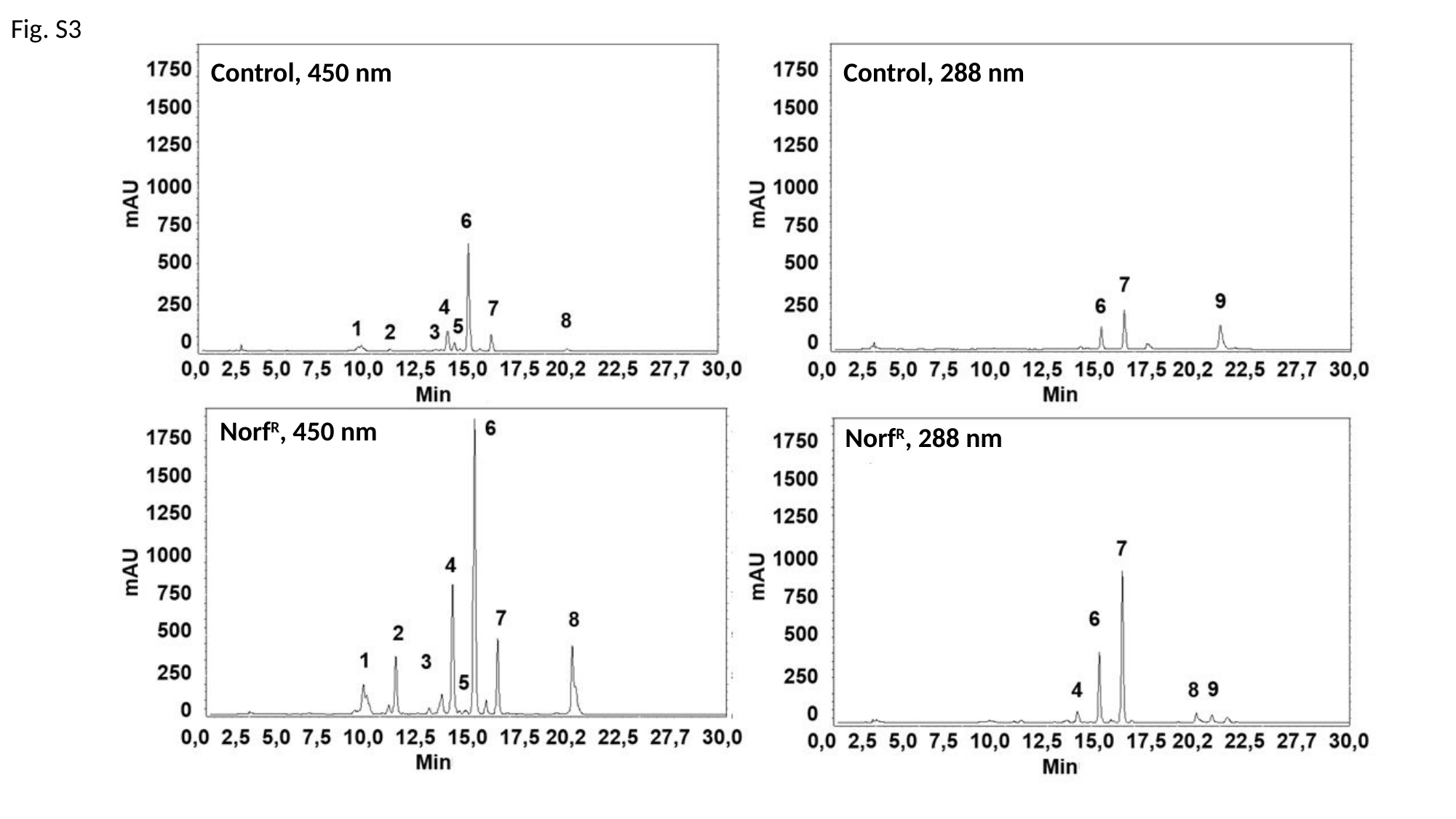

Fig. S3
Control, 450 nm
Control, 288 nm
NorfR, 450 nm
NorfR, 288 nm

## Slide 4
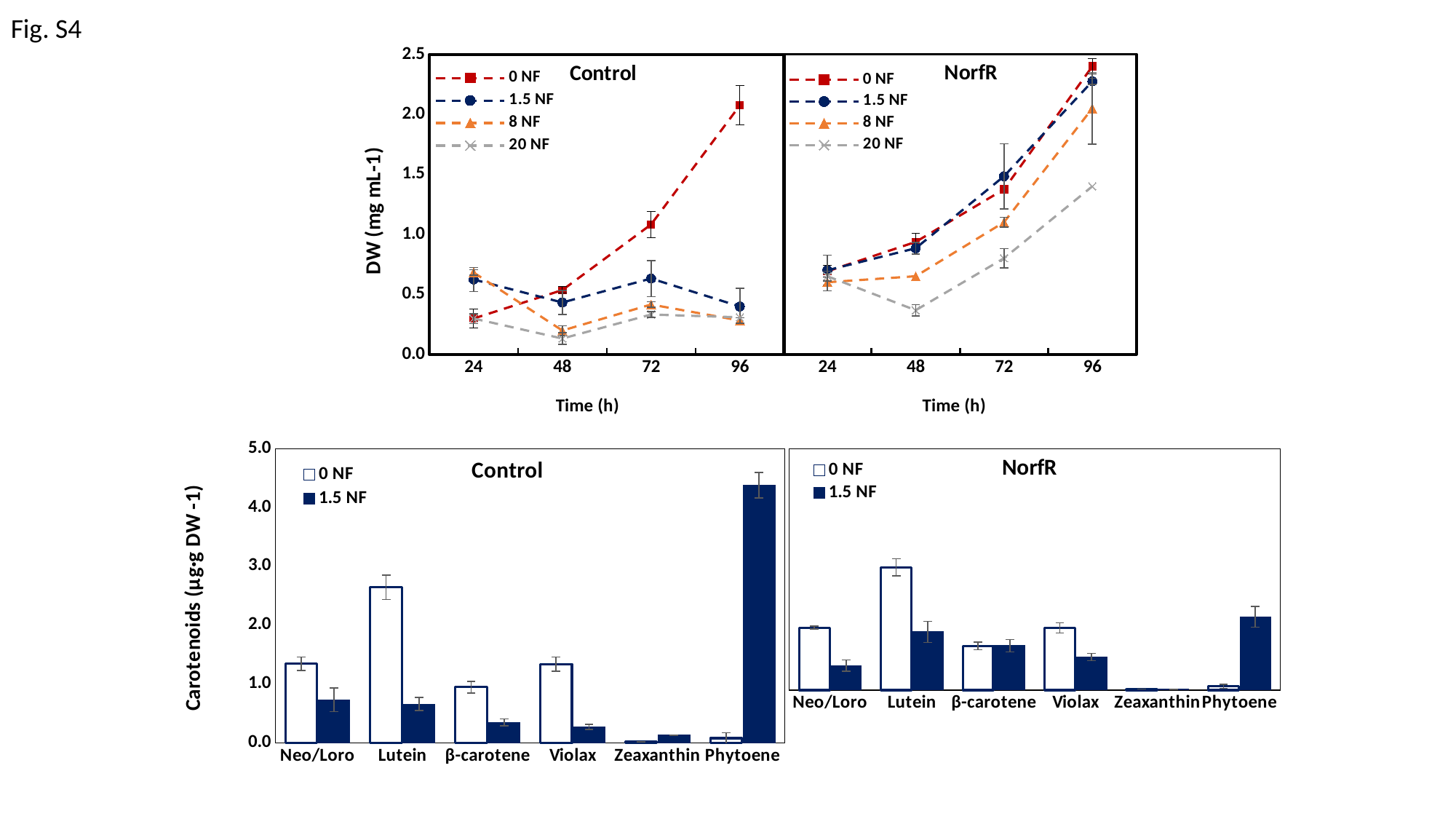

### Chart: NorfR
| Category | | | 8 NF | 20 NF |
|---|---|---|---|---|
| | 0.691 | 0.7000000000000062 | 0.6000000000000172 | 0.6500000000000209 |
| | 0.936 | 0.8833333333333471 | 0.6500000000000025 | 0.3666666666666633 |
| | 1.375 | 1.483333333333318 | 1.0999999999999899 | 0.8000000000000229 |
| | 2.4 | 2.275000000000027 | 2.0499999999999963 | 1.4 |
### Chart: Control
| Category | | | 8 NF | 20 NF |
|---|---|---|---|---|
| | 0.3 | 0.6249999999999589 | 0.6833333333333691 | 0.29999999999996696 |
| | 0.537 | 0.4333333333333596 | 0.20000000000001497 | 0.13333333333335565 |
| | 1.083 | 0.6333333333333376 | 0.4166666666666578 | 0.33333333333333365 |
| | 2.075 | 0.399999999999993 | 0.28333333333330213 | 0.31 |Fig. S4
### Chart: Control
| Category | | |
|---|---|---|
| Neo/Loro | 1.3445959999999493 | 0.730925511368524 |
| Lutein | 2.644474229099174 | 0.6607616951094084 |
| β-carotene | 0.9479099999999642 | 0.34857237348778086 |
| Violax | 1.3394755555555047 | 0.2745737868725929 |
| Zeaxanthin | 0.02 | 0.1332311641680574 |
| Phytoene | 0.08 | 4.37724034234242 |
### Chart: NorfR
| Category | | |
|---|---|---|
| Neo/Loro | 1.2928807692308015 | 0.5098215396825299 |
| Lutein | 2.5427636818262878 | 1.206199977759048 |
| β-carotene | 0.9114519230769458 | 0.9190502559523613 |
| Violax | 1.2879572649572968 | 0.685268397773772 |
| Zeaxanthin | 0.02 | 0.02 |
| Phytoene | 0.08 | 1.5179752261904258 |

## Slide 5
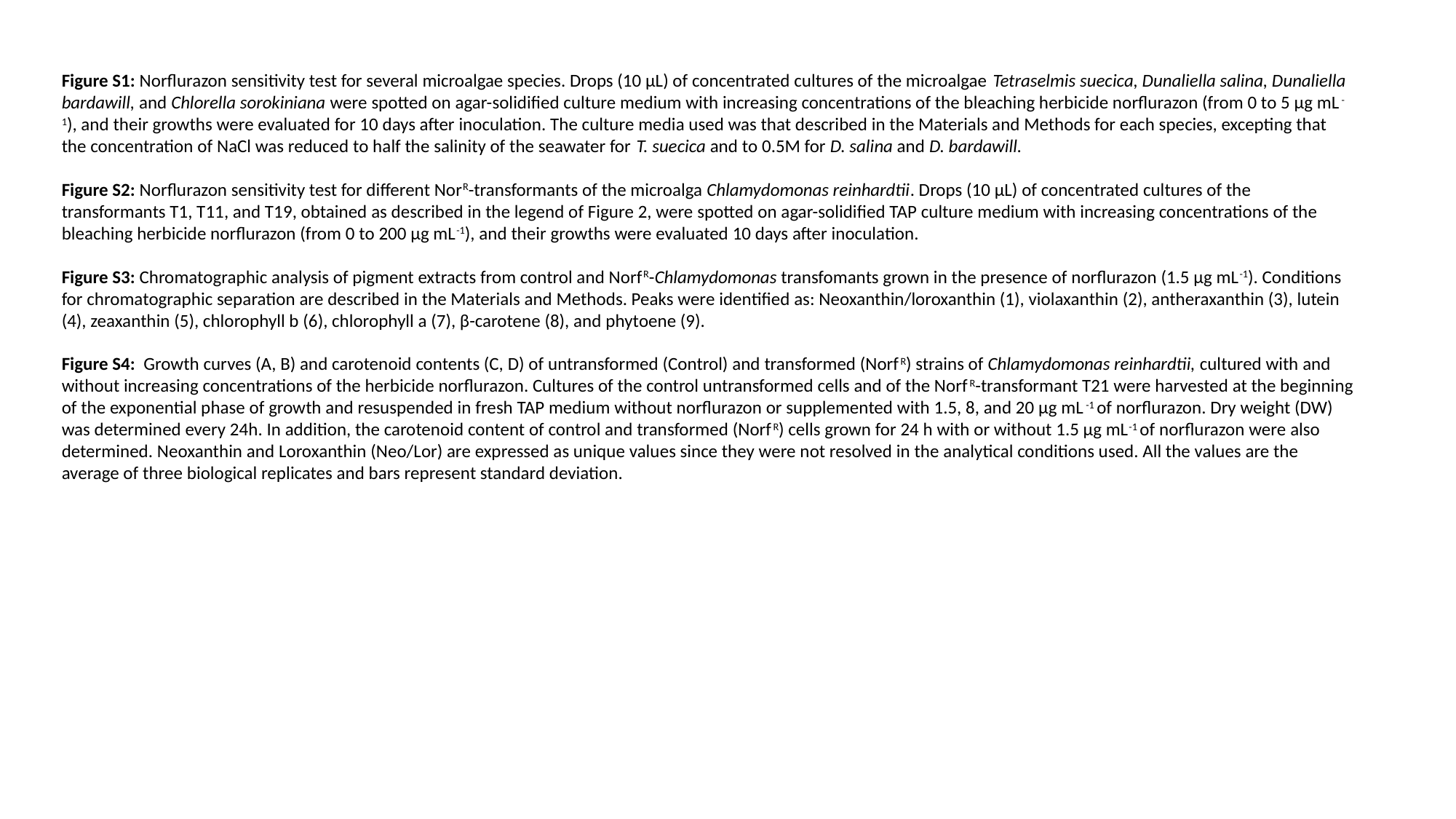

Figure S1: Norflurazon sensitivity test for several microalgae species. Drops (10 µL) of concentrated cultures of the microalgae Tetraselmis suecica, Dunaliella salina, Dunaliella bardawill, and Chlorella sorokiniana were spotted on agar-solidified culture medium with increasing concentrations of the bleaching herbicide norflurazon (from 0 to 5 µg mL-1), and their growths were evaluated for 10 days after inoculation. The culture media used was that described in the Materials and Methods for each species, excepting that the concentration of NaCl was reduced to half the salinity of the seawater for T. suecica and to 0.5M for D. salina and D. bardawill.
Figure S2: Norflurazon sensitivity test for different NorR-transformants of the microalga Chlamydomonas reinhardtii. Drops (10 µL) of concentrated cultures of the transformants T1, T11, and T19, obtained as described in the legend of Figure 2, were spotted on agar-solidified TAP culture medium with increasing concentrations of the bleaching herbicide norflurazon (from 0 to 200 µg mL-1), and their growths were evaluated 10 days after inoculation.
Figure S3: Chromatographic analysis of pigment extracts from control and NorfR-Chlamydomonas transfomants grown in the presence of norflurazon (1.5 µg mL-1). Conditions for chromatographic separation are described in the Materials and Methods. Peaks were identified as: Neoxanthin/loroxanthin (1), violaxanthin (2), antheraxanthin (3), lutein (4), zeaxanthin (5), chlorophyll b (6), chlorophyll a (7), β-carotene (8), and phytoene (9).
Figure S4: Growth curves (A, B) and carotenoid contents (C, D) of untransformed (Control) and transformed (NorfR) strains of Chlamydomonas reinhardtii, cultured with and without increasing concentrations of the herbicide norflurazon. Cultures of the control untransformed cells and of the NorfR-transformant T21 were harvested at the beginning of the exponential phase of growth and resuspended in fresh TAP medium without norflurazon or supplemented with 1.5, 8, and 20 µg mL-1 of norflurazon. Dry weight (DW) was determined every 24h. In addition, the carotenoid content of control and transformed (NorfR) cells grown for 24 h with or without 1.5 µg mL-1 of norflurazon were also determined. Neoxanthin and Loroxanthin (Neo/Lor) are expressed as unique values since they were not resolved in the analytical conditions used. All the values are the average of three biological replicates and bars represent standard deviation.
